# A molecular–mechanical link in shear-induced self-assembly of a functionalized biopolymeric fluid†

**DOI:** 10.1039/d2sm01381a

**Published:** 2023-04-03

**Authors:** Galina E. Pavlovskaya, Thomas Meersmann

**Affiliations:** a Sir Peter Mansfield Imaging Resonance Centre, School of Medicine, University of Nottingham Nottingham NG2 7RD UK galina.pavlovskaya@nottingham.ac.uk +44115 84 68131; b NIHR Nottingham Biomedical Research Centre, Nottingham University Hospitals NHS Trust Queen’s Medical Centre Derby Road Nottingham NG7 2UH UK

## Abstract

^23^Na multiple quantum filtered (MQF) rheo-NMR methods were applied to probe the molecular foundation for flow induced self-assembly in 0.5% *κ*-carrageenan fluid. This method is sensitive enough to utilize an endogenous sodium ion concentration of approximately 0.02%. Rheo-NMR experiments were conducted at different temperatures and shear rates to explore varying molecular dynamics of the biopolymer in the fluid under shear. The temperature in the rheo-NMR experiments was changes from 288 K to 313 K to capture transition of κ-carrageenan molecules from helices to coils. At each temperature, the fluid was also tested for flow and oscillatory shear behaviour using bulk rheometry methods. It was found that the ^23^Na MQF signals were observed for the 0.5% *κ*-carrageenan solution only under shear and when the fluid demonstrated yielding and/or shear-thinning behaviour. At temperatures of 303 K and above, no ^23^Na MQF signals were observed independent of the presence or absence of shear as the molecular phase transition to random coils occurs and the fluid becomes Newtonian.

## Abstract 2

^23^Na multiple quantum filtered (MQF) rheo-NMR methods were applied to probe the molecular foundation for flow induced self-assembly in 0.5% *κ*-carrageenan fluid.

## Graphical abstract

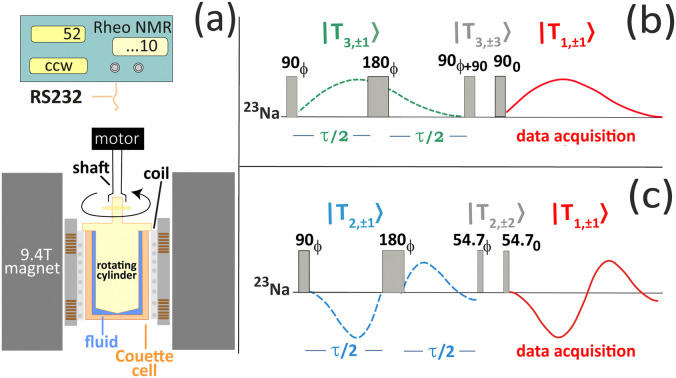

## 1 Introduction

The mechanical properties of many non-Newtonian fluids are determined by the existence of specific short range intra- and intermolecular arrangements making fluids nano- or micro-structured. In polymeric fluids, these molecular arrangements might be created over different length scales involving various interactions within, and between, not only the macromolecules themselves, but also the interactions of the biomolecules with solvent molecules, ions, and other possible constituents of biopolymeric fluids. Most common examples of such interactions in polymeric fluids are hydrogen bondings, electrostatic interactions and dispersive forces, often resulting in intra- and intermolecular chain overlaps or chain cross-links in solutions of polymers thereby affecting the rheology of the fluids.^1^ Moreover, changes in the molecular conformation greatly affect the mechanical properties of the fluids, and these changes, specific to a particular biopolymeric system, can be induced by varying its ionic strength and temperature.^1–5^

*κ*-Carrageenan is a linear sulphated anionic polysaccharide composed of repeating units of 1,3 linked β-d-galactopyranose and a 1,4 linked 3,6-an-hydro-α-d-galactopyranose. It is produced by extraction from edible red seaweeds^6^ and used in food sciences,^7,8^ pharmaceutical industry,^9^ biotechnology,^10^ tissue engineering^11^ and medical applications.^12^ In aqueous solutions and in the presence of compensating metal cations, *κ*-carrageenan molecules change their conformation upon temperature variation.^1,13^ At temperatures lower than ambient temperature, *κ*-carrageenan molecules exist in the double helix conformation and may form a network by an intermolecular synergetic interaction with neighbouring molecules.^13,14^ When the temperature increases to the ambient temperature range, this synergy breaks down due to the increased molecular motion and *κ*-carrageenan molecules exist as separated single helices in the solution. Upon further temperature increase, *κ*-carrageenan helices start to unfold and transform into random coils at around 310 K and above. Interestingly, the transition temperature region shifts depending on the concentration and nature of the compensating cations but not on the concentration of the polymer itself.^2^ The increase in the cation concentration shifts the range to higher temperatures;^15^ however, sodium as a compensating cation has the weakest influence on the shift of the transition temperature.^4^ The transition process is thermally reversible and is quite stable through the entire heating/cooling ↔ cooling/heating cycle.^14,16^ The helix to random coil transition is found in the majority of *κ*-carrageenan systems, and manifests itself in the gel–sol transition that accompanies a distinct change in material functions.^2,5,17^ Controlling of the transition can be used to selectively enhance the mechanical properties of *κ*-carrageenan based materials where appropriate. Such capabilities are potentially of great importance as *κ*-carrageenan containing systems are attractive for tissue engineering because of their ability to form bio-compatible hydrogels.^11^

Additive manufacturing is often performed at temperatures that are different from operational temperatures of the final products.^18^ Examples of growing importance are the design and production of tissue implants.^19^ Hence, there is an industrial demand for non-intrusive labels that could be used to monitor the *in situ* mechanical properties of final tissue implants manufactured using *κ*-carrageenan, especially at varying temperatures. Most of the *κ*-carrageenan containing tissue implants would naturally contain Na^+^ cations, usually at a physiological concentration. Hence, Na^+^ can be used to track changes in the mechanical properties of these materials using ^23^Na magnetic resonance spectroscopy (MRS) and even ^23^Na magnetic resonance imaging (MRI) if the sodium concentration is sufficiently high.

Rheo-NMR allows one to study molecular responses to deformations and to correlate these responses with the mechanical properties of fluids determined through bulk rheology methods.^20–22^ The well-established rheo-NMR methodology usually uses proton detection, for example in protein solution studies,^23,24^ and sometimes additional labels like deuterium^25^ have been introduced to explore more fundamental soft condensed matter phenomena, for example shear banding in micellear^26^ and other systems.^27,28^ Rheo-NMR can also be used for sodium detection and it has been shown that multiple quantum filtered (MQF) sodium (^23^Na) methods can be used to observe the molecular order created in biofluids in shear changing zones under flow.^29^

In this work, we applied ^23^Na MQF methods in combination with rheo-NMR to monitor the shear-induced molecular order that occurs in the 0.5% *κ*-carrageenan fluid during the temperature ramp as *κ*-carrageenan molecules transition from helices to random coils. Molecular responses monitored using ^23^Na MQF methods were further matched to the change in material functions of the 0.5% *κ*-carrageenan fluid characterised by shear and oscillatory rheometry. Note that the outcomes of this study have great potential to impact *in vivo* studies of biopolymeric fluids with ^23^Na whole body MRS/MRI at a body physiological sodium concentration that exceeds the ^23^Na concentration used in this work by approximately 40 times.

## 2 Experimental section

### Materials

The Na^+^ form of *κ*-carrageenan was purchased from Sigma-Aldrich, UK, and used without further purification. An endogenous sodium concentration of 0.02%, as estimated from the equation in the study of Gobet *et al.*,^30^ was used in the present work and no additional sodium was added during fluid preparation. Three separate batches of 50 ml of the 0.5% *κ*-carrageenan fluid were prepared by dissolving 0.25 g of the polymer in 50 g of distilled water. During stirring, solutions were covered with parafilm and heated to 333 K to ensure complete dissolution of the added polymer. Traces of sodium azide were added to all solutions to avoid bacteriological contamination. After dissolution, solutions were placed in closed vials, caps were sealed with parafilm to avoid evaporation during storage and vials were stored at room temperature.

### Rheometry

An AR-G2 (TA Instruments, UK) strain-controlled rheometer was used to collect the shear and oscillatory rheology data in this study. Standard TA Instruments Couette geometry was used to collect the data to match bulk rheology to rheo-NMR outcomes. Shear rheology was performed using a stepped flow procedure at 288 K, 295 K, 303 K and 313 K. Shear rates were varied from 0.0001 s^−1^ to 300 s^−1^. Amplitude sweeps were performed at 283 K, 288 K, 295 K and 303 K using the 0.01% to 1000% strain range at 6.28 rad s^−1^. Frequency sweeps were performed at 283 K, 288 K, 295 K and 303 K in the 0.1 to 100 rad s^−1^ radial frequency range at 1% strain. Temperature sweeps were performed at 1% strain from 283 K to 310 K. Fluids were first cooled to 283 K, and left at this temperature until solutions were temperature equilibrated and then heated to 313 K at a 2 K min^−1^ rate. The equilibrium was confirmed using the instrument built-in functions and only after this the data collection was performed. At each temperature, frequency sweeps were performed in the frequency range from 0.1 to 100 rad s^−1^. In a separate temperature ramp, shear viscosity was measured at constant *γ̇* = 10 s^−1^. Temperature was changed at a 5 K min^−1^ rate from 283 K to 313 K. Data were analysed using TA Instruments TRIOS software, v 4.4.0.41651. More details on data sampling and replicated measurements are provided in the ESI† file.

### Rheo-NMR

**Fig. 1:**
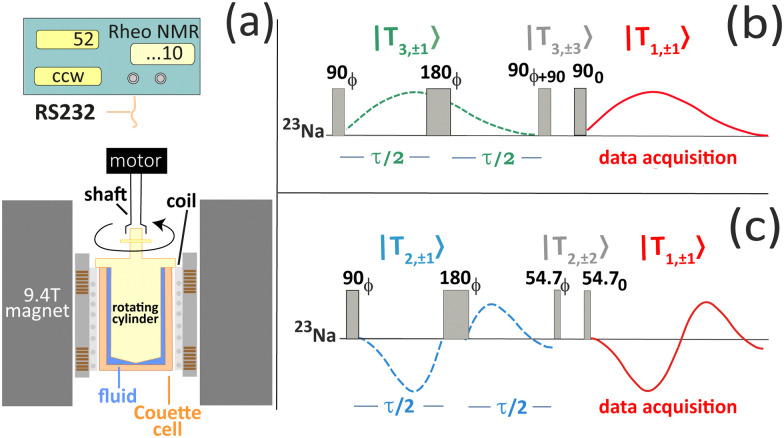
(a) The outline of the rheo-NMR experimental set up. Time diagrams of ^23^Na multiple quantum filtered (MQF) (b) triple quantum filtered (TQF) and (c) double quantum filtered (DQF) magic angle (MA) pulse sequences used in this study. The TQF sequence of ^23^Na (Spin 
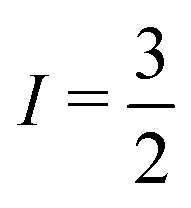
) utilizes the |*T*_3,±1_〉 coherence that is generated through quadrupolar coupling in a molecular alignment phase or, alternatively, through quadrupolar relaxation processes caused by the slow molecular motion. This coherence cannot be detected directly but is transformed briefly into the triple quantum coherence, |*T*_3,±3_〉, for the TQF process that leaves only |*T*_3,±1_〉 at the beginning of the data acquisition. The same molecular processes that generated this coherence will also reconvert it back into the observable |*T*_1,±1_〉 coherence that emerges over time. Similarly, the DQF MA sequence utilizes |*T*_2,±1_〉. However, this coherence can only be caused by coupling and not through relaxation. The oscillatory |*T*_2,±1_〉 coherence is therefore an indication for the presence of the molecular alignment. Single quantum experiments (not depicted here) utilize a single 90 degree pulse followed by a typical free induction decay (FID) of the observable |*T*_1,±1_〉 coherence. Numbers and symbols displayed in the rheo-NMR unit are as follows: ‘52’ are rotations per minute, ‘ccw’ is the direction of shearing, which is counterclockwise in this experiment and ‘…10’ is the gear ratio.

Rheology experiments inside the 9.4T super-conducting magnet were performed using a commercially available rheo-NMR attachment and a non-magnetic Couette cell produced by Bruker (Germany). The Couette cell, machined from PEEK, has an outside diameter of 19 mm, with a bob diameter of 18 mm resulting in a 1 mm gap between the stationary wall and the rotating inner bob. The Couette cell was inserted inside a 25 mm resonator (Bruker, Germany) tuned to a ^23^Na resonance frequency of 105 MHz and mounted into the centre of the 9.4T magnet. The width of the π/2 sodium pulse was 52 μs. A variety of shear rates were provided using an externally located motor, remotely controlled through built-in scanner software. The coupling of the rheo-cell to the motor was achieved using a drive shaft inserted into the magnet bore. The outline of the setup is shown in Fig. 1(a). Shear rates of 11.6 s^−1^, 29 s^−1^, 58 s^−1^ and 87 s^−1^ were used for the ^23^Na NMR experiments conducted at temperatures of 288 K, 295 K, 303 K and 313 K. The temperature in the Couette cell was changed by changing the temperature in the gradient coil chiller unit. The temperature in the gradient coils was read from the built-in sensor attached to the gradient amplifier control console. The temperature in the Couette cell was further checked using a thermocouple attached in the bottom of the Couette cell. The reading of the thermocouple was within ±0.5 K of the temperature read from the gradient console.

### ^23^Na multiple quantum filtered (MQF) spectroscopy

^23^Na MQF spectroscopy was performed using the triple quantum filtered (TQF) sequence shown in Fig. 1(b) and the double quantum filtered (DQF) sequence shown in Fig. 1(c). The latter one includes 57.4 degree (magic angle) r.f. pulses to filter out all coherences except for the |*T*_2,±1_〉 coherence (*i.e.* the DQF MA sequence). Note that in the biopolymeric systems studied, the |*T*_2,±1_〉 coherence is only generated when some kind of ordered phase is present that has a net alignment with respect to the magnetic field. The net alignment causes a quadrupolar coupling in the nuclear spin 
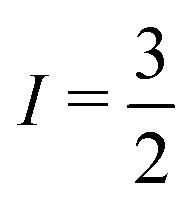
 system of ^23^Na, that, in turn, causes the |*T*_2,±1_〉 coherence to be present. Therefore, non-zero signals obtained from the DQF MA sequence indicate the molecular alignment. See ref. 31 and 32 for a more detailed discussion of this fundamental physical effect utilized in this work. The 48-step and 36-step phase cycling for TQF and DQF MA, respectively, were used to achieve optimal SNRs. The width of the ^23^Na π/2 pulse was 83 μs at 200 W. The |*T*_2,±1_〉 and |*T*_3,±1_〉 coherences are generated during the echo time *τ* and 45 increments of the *τ* time were used to monitor the time evolution of these coherences in the MQF experiments in the presence and absence of varying applied shear fields. Data were collected in 2048 data points for each *τ* time, and with a 100 ms recycle delay. 960 and 1024 transients were collected at each *τ* value and resulted in 3 h 9 min and 3 h 21 min for full TQF and DQF MA time evolution experiments, respectively. Error bars were evaluated from signal-to-noise ratios (SNRs) obtained for each individual spectrum at each *τ* value using well-established procedures.^33^

## 3 Results and discussion

### Shear and oscillatory rheology

**Fig. 2:**
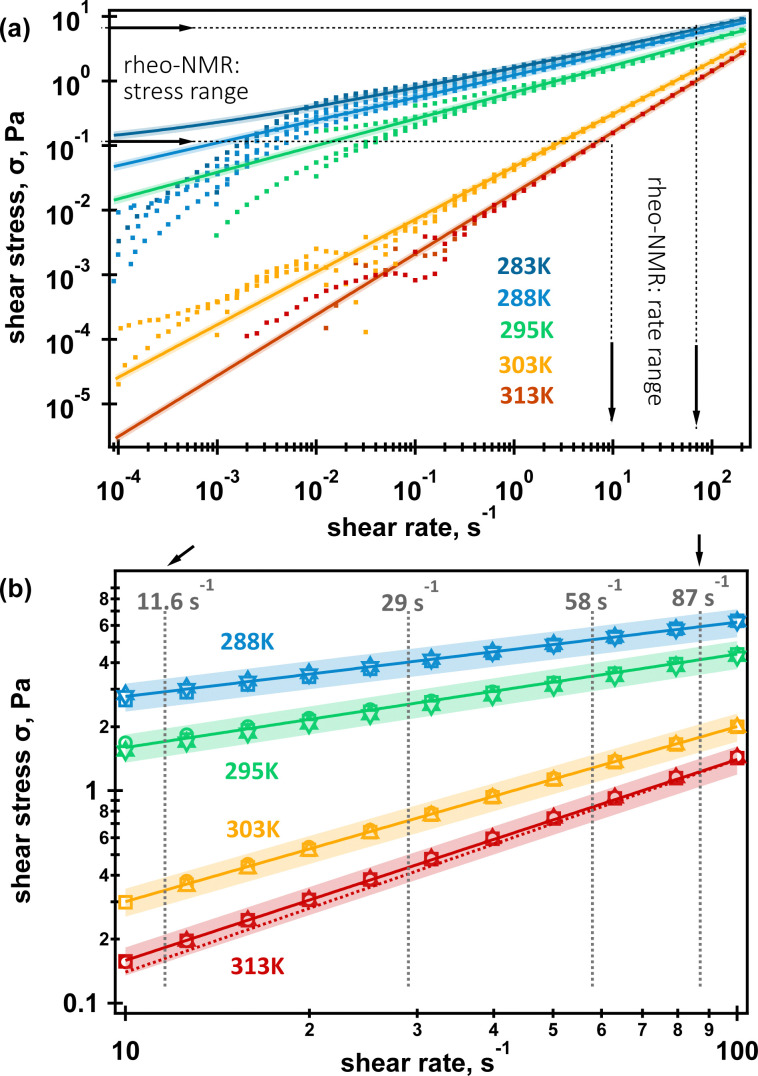
Shear stress *vs.* shear rates of the 0.5% aqueous *κ*-carrageenan fluid at different temperatures: (a) full range of shear rates and temperatures probed in this work. Dashed lines with arrows indicate shear stress and shear rate ranges used by the ^23^Na MQF rheo-NMR; (b) ^23^Na MQF rheo-NMR shear stress *vs.* shear rate ranges for four temperatures. Dots in (a) represent multiple shear stress curves collected in different shear rate ranges at a given temperature. These replicated experiments are shown in symbols in (b) for each temperature. Solid lines represent the predicted shear stress *vs.* shear rate curves using averaged parameters displayed in Table 1. Shading represents a 15% deviation from the predicted behaviour. The dashed line in (b) is the Newtonian behaviour. All rheo-NMR data were collected in the shear-thinning range.

The shear rheology of the 0.5% aqueous *κ*-carrageenan fluid is displayed in Fig. 2(a). The fluid was tested at shear rates from 10 s^−4^ to 200 s^−1^ at 283 K, 288 K, 295 K, 303 K and 313 K. Shear stress *vs.* shear rate curves were analysed using the built-in “stress *vs.* rate” routine in TRIOS software first, and the best model was applied to extract relevant model parameters for each replicated measurement. The extracted parameters for each replicated measurement were grouped and further analysed with the WaveStats built-in function in IGORPro8 to result in the averaged value and the standard deviation in each group. If a parameter standard deviation exceeded its averaged value, then the next best model was used to produce the averaged parameters using the same workflow. Further details on the analysis workflow are found in Fig. SI1, ESI,† and the averaged parameters for each tried model are shown in Table 1. It was found that both the Herschel–Bulkely and Power Law models described well fluid behaviour at 283 K. As the standard deviation was within 30% of the averaged yield stress, the fluid was treated as a yield stress fluid at this temperature. The standard deviations at 288 K and 295 K exceeded the averaged yield stresses by three and two times, respectively. Therefore, the Herschel–Bulkely model was decided to be statistically insignificant; therefore, the fluid was treated as a Power law fluid up to 313 K. The Power law model described the experimental behaviour of the fluid at 313 K very well, as can be seen from both Table 1 and Fig. 2(b); however, the averaged rate index *n* = 0.946 was very close to that of 1. Therefore, the Newtonian model was also tried for the fluid at this temperature. This resulted in a smaller error for the Newtonian viscosity as can be seen from Table 1 but the Newtonian model deviated more from the experimental points as can be seen in Fig. 2(b). Nonetheless, the Newtonian behaviour was within 15% of the Power law model. Considering that the *n* index was almost unity when the Power law model was used, it was decided that the Newtonian model was an appropriate model for the fluid at 313 K. Fig. 2(b) further shows that all rheo-NMR experiments were conducted in the stress range when a well-defined fluid model, namely a Power law one and a Newtonian one, can be used to describe fluid's flow behaviour. The temperature range used in this study also covered a variety of molecular arrangements formed in this fluid by *κ*-carrageenan molecules upon heating.^2,5,17^ This can be correlated to the changes in the rate indices obtained that further confirm that the fluid demonstrates shear-thinning behaviour up to 303 K (*n* < 1) and then shifts to predominantly Newtonian behaviour at 313 K as the rate index *n* is approaching unity, see Table 1.

**Table 1:** Averaged parameters using Herschel–Bulkley (HB), Power law (PL) and Newtonian (N) models extracted after the analysis of multiple stress *vs.* rate curves displayed in Fig. 2 at all temperatures studied

*T*, K	Yield stress, Pa	Consistency/viscosity, Pa. s	*n*, rate index
283 K			
(HB)	0.06 ± 0.02	1.49 ± 0.05	0.35 ± 0.03
(PL)		1.56 ± 0.07	0.322 ± 0.001
288 K			
(HB)	0.02 ± 0.07	1.23± 0.08	0.36± 0.01
(PL)		1.24 ± 0.08	0.35 ± 0.01
295 K			
(HB)	0.1 ± 0.2	0.49 ± 0.04	0.47 ± 0.01
(PL)		0.58 ± 0.06	0.44 ± 0.02
303 K			
(PL)		0.045 ± 0.002	0.824 ± 0.006
313 K			
(PL)		0.018 ± 0.001	0.946 ± 0.002
(N)		0.0140 ± 0.0001	

To determine the linear viscoelasticity region (LVR) in the *κ*-carrageenan fluid at temperatures used in rheo-NMR, the amplitude sweep tests were performed. The results of these tests are displayed in Fig. SI2, ESI.† It was determined that the LVR region persisted up to *γ*_LVR_ = 20% and up to 303 K. It was not possible to perform an amplitude test at 313 K because of the noise. In the LVR, the storage modulus *G*′ was larger than the loss modulus *G*′′ at 283 K, 288 K and 295 K probably indicating the more structural presence in the *κ*-carrageenan fluid in this temperature range. This was probably associated with the synergetic network behaviour of semi-rigid rods as previously has been reported for *κ*-carrageenan solutions of similar concentrations.^14^ At 303 K, *G*′′ becomes dominant in the LVR which most likely is associated with *κ*-carrageenan molecules transitioning from helices to coils as previously was observed for *κ*-carrageenan upon the increase in temperature.^16^

**Fig. 3:**
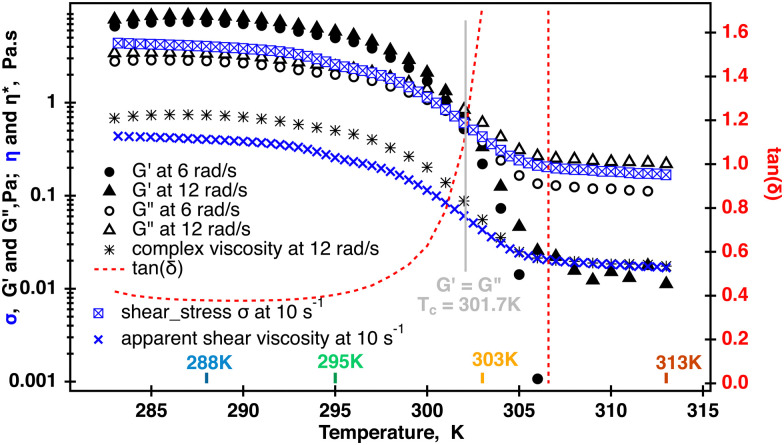
Temperature sweep of the 0.5% aqueous *κ*-carrageenan fluid. Dynamic moduli and shear stress were measured in two separate temperatures sweeps. Dynamic data displayed for 6 and 12 rad s^−1^ were collected at 1% strain. One point complex viscosity was computed from the 12 rad s^−1^ data. Crossover was determined using TRIOS and occurred at *T*_c_ = 301.7 K. Shear stress was measured at a constant shear rate of 10 s^−1^ and used to calculate one point shear viscosity at each temperature. The complex viscosity and shear viscosity were the same when *G*′ became insignificant. The symbol size in each curve roughly represents the spread range from an averaged value at each temperature point.

Transitioning of *κ*-carrageenan molecules from helices to coils was confirmed by two separate temperature sweeps where both the apparent shear viscosity and the modulus were measured at each point temperature as shown in Fig. 3. The shear viscosity measured at *γ̇* = 10 s^−1^ decreased approximately two orders of magnitude during the temperature ramp. As has been shown previously, a similar drastic change in the viscosity during a temperature ramp in *κ*-carrageenan systems was associated with the helix to coil transition as confirmed by optical rotation experiments conducted in parallel.^14^ At the same time, data shown for two radial frequencies at 1% strain demonstrate that for up to 301.7 K the storage modulus *G*′ dominates over the loss modulus *G*′′. At temperatures above 301.7 K, the loss modulus becomes dominant with the storage modulus becoming undetectable at temperatures above 305 K. Tan(*δ*) starts to increase from at right above 295 K approaching infinity from 305 K. The complex viscosity also starts to decrease at 295 K and reaches a plateau at above 305 K. Moreover, the complex viscosity measured at *ω* = 12 rad s^−1^ overlays the apparent shear viscosity at *γ̇* = 10 s^−1^ at a temperatures above 305 K, and as the Cox–Merz relationship holds at a single value of the radial frequency and a shear rate. This is an indication that the fluid becomes a semi-diluted polymer solution with no entanglements between the molecules. In this case, it appears that the loss of the elastic response also coincides with the helix to coil molecular transition in these *κ*-carrageenan systems during the temperature ramp. This has been previously reported and confirmed by optical rotation^2^ and calorimetric^34^ methods for similar *κ*-carrageenan systems.

**Fig. 4:**
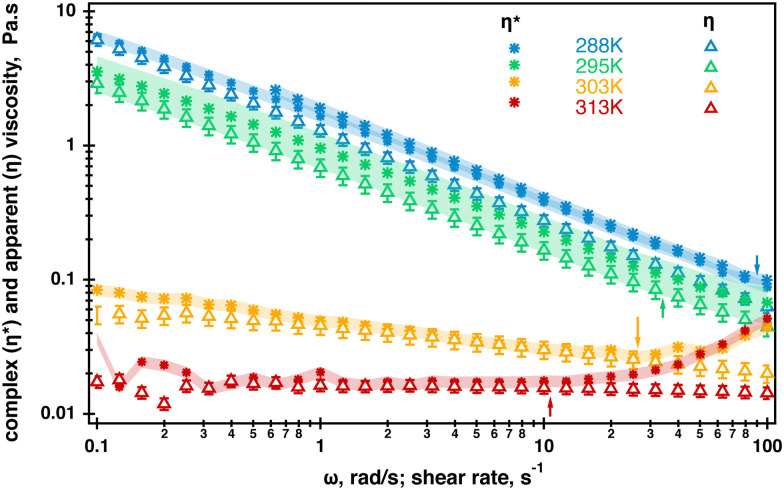
Overlay of the complex viscosity (stars) and apparent viscosity (open triangles) of the 0.5% aqueous *κ*-carrageenan fluid in the temperature range used in ^23^Na rheo-NMR experiments. The Cox–Merz relationship starts to hold at 303 K. Arrows indicate the onset of inertia effects occurring at each temperature during oscillatory measurements as the raw phase becomes larger than 150^0^. Error bars in the apparent viscosity data represent a 10% deviation from an averaged value computed at each temperature. The shaded area in the complex viscosity data represent 10% at 288 K, 303 K and 313 K. The shaded area was increased to 30% at 295 K because of the lower data quality (Fig. SI4, ESI†). The complex viscosity data at 313 K were extracted from the multiple frequency collected during oscillation temperature sweeps and are displayed in Fig. 3 as no oscillatory rheology is collected at 313 K.

Extended frequency range sweeps were measured in separate experiments and their results are displayed in Fig. SI4, ESI.† No crossover was measured within the range of probed radial frequencies. Unfortunately, the quality of the data did not allow for a meaningful relaxation analysis; therefore, the complex viscosity was evaluated from the raw data in the temperature range probed by rheo-NMR using the TRIOS built-in Cox-Merz transformation. Where possible, average values from replicated measurements were computed and the results are displayed in Fig. 4. As can be seen from Fig. 4, the differences between the complex viscosity and apparent viscosity are small. However, the complex viscosity is most likely larger at 288 K which could be an indication of molecular entanglements present in the fluid at this temperature.^5^ It was difficult to determine whether the Cox–Merz relationship holds at 295 K because of the quality of the data; however, it might be a hint of the closer compared to the 288 K overlap. At 303 K and 313 K, the complex viscosity and apparent viscosity match within 10% which is probably an indication of the break up of molecular entanglements in the fluid. This is probably caused by the molecular transition from helices to random coils resulting in the fluid behaving as a semi-dilute polymer solution. It is also worth noting that the complex viscosity starts to increase and the onset of the increase is shown by arrows in Fig. 4. According to the specification of the TA Instruments for ARG2 rheometers, this is most likely associated with inertia effects occurring in oscillatory experiments when the raw phase exceeds 150°. This could be negated by changing to a lower weight geometry but was not performed in this work. In all, better quality data are required for more definitive conclusion but our data are an indication that the helix–coil transition most likely occurred in the fluid.

**Fig. 5:**
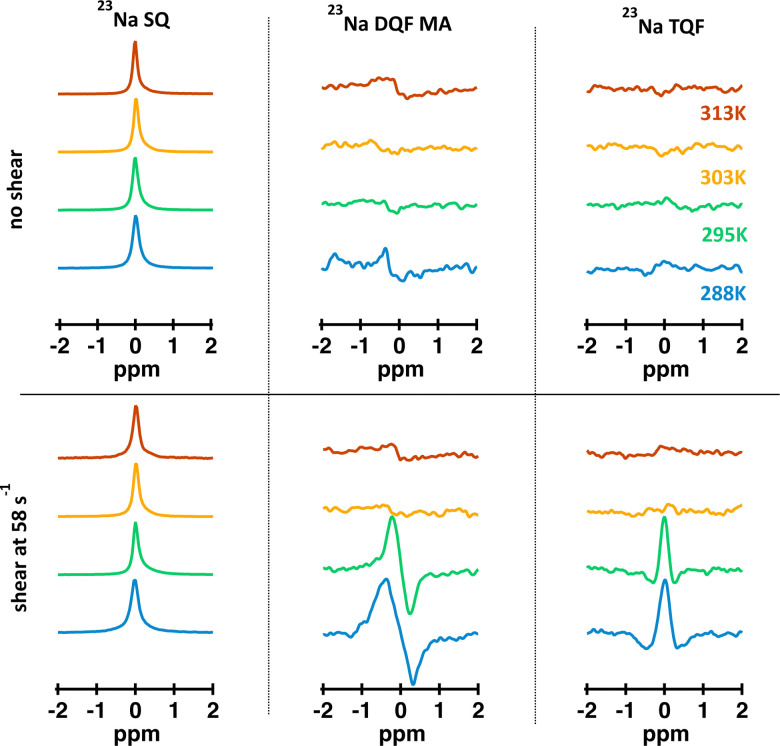
^23^Na SQ, DQF MA and TQF spectra of the 0.5% aqueous *κ*-carrageenan solution measured at different temperatures in the Couette cell at *γ̇* = 58 s^−1^ and in the absence of shear.

Such a drastic change in the molecular conformation will have an effect on the molecular dynamics of sodium cations. ^23^Na single quantum (SQ) and multiple quantum filtered (MQF) signals were measured in the Couette cell while the fluid sample was sheared at *γ̇* = 58 s^−1^ and in the absence of shear at 288 K, 297 K 303 K and 313 K. The resulting spectra are displayed Fig. 5. Temperature-dependent shear and oscillatory rheology displayed in Fig. 3 indicate three distinct regions marked in fluid behavior. The first region, where the storage modulus dominates over the loss modulus up to 295 K, the second one, or a transitional region, where the modulus crossover occurs at 301.7 K, and the third one, or an isotropic region, where the fluid behaves as a pure liquid (loss of *G*′) and becomes Newtonian. This corresponds to the change in the conformation of *κ*-carrageenan molecules in solution from helices (at 288 K) to random coils (at 313 K). Sodium spectra obtained using single quantum (SQ), or one pulse, spectroscopy at *γ̇* = 58 s^−1^ at the temperatures selected from these three regions appear very similar to the ones recorded in the absence of the shear as seen in Fig. 5 (left panel). However, sodium DQF MA spectra shown in Fig. 5 (central panel), are observed only at temperatures 288 K and 295 K corresponding to the range where *G*′ dominates and *κ*-carrageenan molecules preserve their helix conformation when sheared. Interestingly, when at rest, DQF MA signals are absent. The absence of DQF MA signals in the absence of shear at these temperatures is an indication that the helices are randomly oriented in the fluid and hence do not produce any aligned phase. Since the DQF MA sequence detects sodium signals only from the ordered phases, this implies that there is a shear-induced ordered phase created by *κ*-carrageenan helices as they adapt to the applied shear, Fig. 5 (central panel). The formation of the shear-induced ordered phase was not detected when *κ*-carrageenan molecules were in the random coil conformation as evident from the absence of DQF MA signals at temperatures 303 K and above. TQF signals displayed in Fig. 5 (right panel) follow a similar trend to DQF MA signals. In principle, TQF sodium signals can be observed in the absence of an ordered environment; however, in this system, TQF signals followed the same trend as DQF MA signals. Therefore, TQF signals were also recorded only from the ordered environment created by *κ*-carrageenan helices in the shear field.

Molecular insights into the formation of the shear-induced ordered phase were further gleaned using the time dependence of the build-up of ^23^Na MQF rheo-NMR signals at a variety of shear rates and temperatures. To ensure that the maximum of the sodium signal build up was reached in each experiment, ^23^Na MQF spectra were collected using the timing diagrams displayed in Fig. 1(b and c) with 45 time increments in the *τ* time in the presence and the absence of the applied shear field at different temperatures. The obtained data sets were interpreted using well-known equations that describe the time evolution of MQF signals as a function of the multiple quantum creation time, *τ*.^35^ To reduce the number of fitting parameters, DQF MA signals were analysed first using the following equation:^35^1
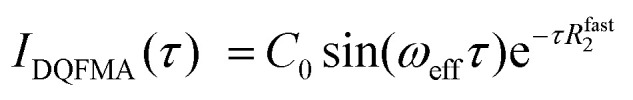
where *C*_0_ is the amplitude of the DQF MA signal; *ω*_eff_ is the residual quadrupolar coupling constant (QCC) in Hz and is a measure for the anisotropy, or alignment, within the biopolymeric fluid; *τ* is the double quantum coherence creation time, s; and *R*^fast^_2_ is the fast component of sodium signal transverse relaxation, s^−1^. The obtained values of *ω*_eff_ and *R*^fast^_2_ were used as non-adjustable coefficients in analysing the ^23^Na TQF signal time evolution according to:^35^2
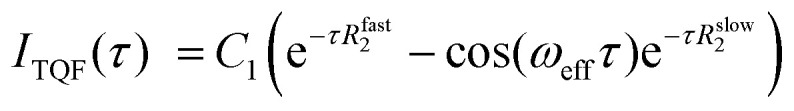
where *C*_1_ is the amplitude of the TQF signal and *R*^slow^_2_ is the slow component of the sodium signal transverse relaxation, s^−1^, and the meaning of other parameters is the same as in the description to eqn (1). This approach is well established and has been used before.^32,35^

**Fig. 6:**
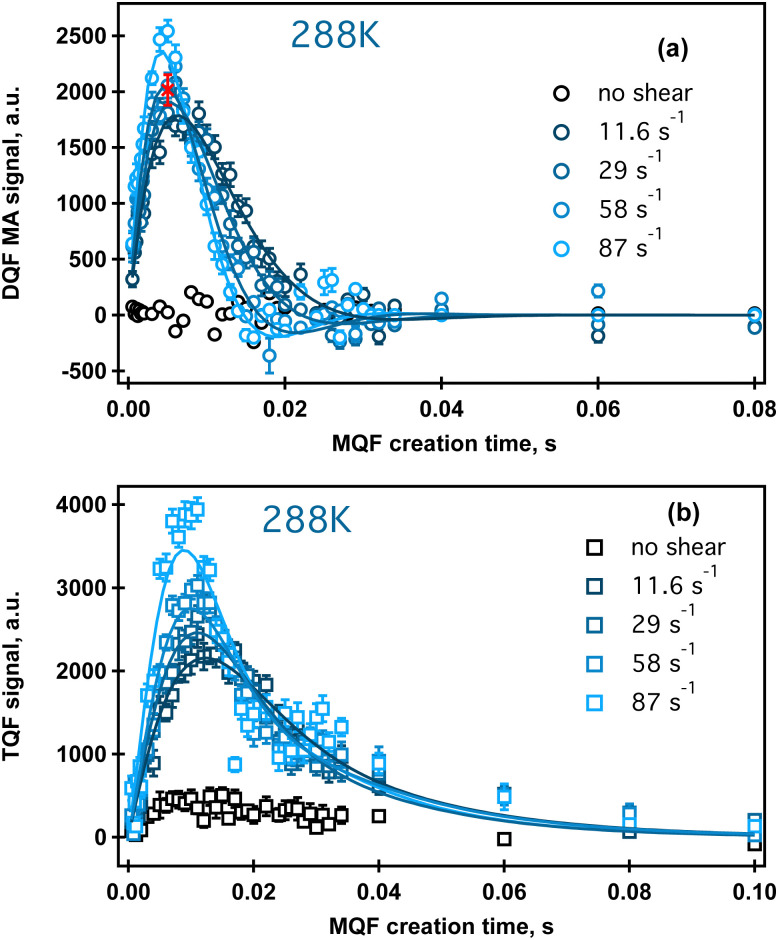
Rheo-NMR data analysis at 288 K: ^23^Na DQFMA (a) and ^23^Na TQF (b) time evolution at different shear rates. Solid lines represent fitting to eqn (1) and (2), respectively, as described in the text. Fitting parameters and errors are displayed in Table 2. Data collected in the absence of shear are shown in black symbols. Red cross in (a) shows a replicated ^23^Na DQF MA experiment at *γ̇* = 58 s^−1^ and *τ* = 5 ms. Replicated spectra are shown in Fig. SI4, ESI.†

The results of this data analysis for the ^23^Na MQF rheo-NMR experiments collected at 288 K at different shear rates indicated in Fig. 2 are shown in Table 2 and are displayed in Fig. 6.

**Table 2:** Relevant fitting parameters extracted after analysing the 288 K data displayed in Fig. 6 using eqn (1) and (2), please refer to Table SI3, ESI, for details on other fitting parameters

*γ̇*, s^−1^	*ω*_eff_, Hz	*R* ^fast^_2_, s^−1^	*R* ^slow^_2_, s^−1^
11.6	111 ± 7	135 ± 8	46 ± 3
29	142 ± 7	145 ± 8	52 ± 3
58	188 ± 6	153 ± 9	46 ± 3
87	213 ± 7	170 ± 10	53 ± 5

It has been suggested that at this temperature, *κ*-carrageenan molecules exist in the helix conformation and might have a tendency to form a network by a synergetic chain overlap with the neighbouring molecules.^4^ One could assume that the networking should contribute to a preferential molecular order in the fluid that should be detected using the ^23^Na MQF spectroscopic methods. However, as can be seen from Fig. 5 (upper panel, spectra shown in blue) and Fig. 6 (a and b, black cross symbols), no MQF sodium signals were detected in the absence of shear. The absence of ^23^Na DQF MA signals when no shear is applied as shown in Fig. 5 and 6 provides evidence that there is no well-defined ordered phase with a preferential orientation with the respect to the external magnetic field that can be detected by sodium ions. The sodium ions probe an ordered phase through the distortion of the electron shell symmetry that interacts with the electric quadrupole moments of the sodium nuclei. Over the time course of the NMR scan, this may result a non-zero value of the residual quadrupolar coupling constant *ω*_eff_. Note that a non-zero quadrupolar coupling *ω*_eff_ in eqn (1) is needed for any DQF MA signals to be formed. The time averaged interactions experienced by Na^+^ ions in the aqueous solution typically do not lead to a net quadrupolar coupling over the NMR relevant time scale. A persistent non-zero coupling of the nuclear electric quadrupolar moment with the sodium electron shell can however be caused by an anisotropic molecular environment that breaks the spherical symmetry of the sodium ions. An anisotropic phase is imposed by molecules that display some kinds of net alignments rather than a random order. In the present case, strong ^23^Na DQF MA signals are an indication of the molecular order formed at the onset of shear. This implies a shear induced phase with a likely structure that is probably aligned with the flow direction. These shear induced ordered domains are most likely localised to the shear changing zones that may be formed within the flowing fluid inside the gap of the Couette cell. In a previous work, the existence of such a localized, aligned phase was proven in the biopolymeric flow through a cylindrical tube, utilizing DQF MA contrast in the ^23^Na MRI in physiological sodium concentration.^29^ Unfortunately, because of the low sodium concentration in the present system (0.02% as estimated using ref. 30) sodium imaging was not feasible and was not performed.

The TQF results displayed in Fig. 6(b) provide further insights into molecular dynamics influenced by shear probed through the NMR properties of sodium ions. Strong ^23^Na TQF signals, Fig. 6(b), are observed only in the presence of shear. Meanwhile, DQF MA signals require a non-zero quadrupolar coupling *ω*_eff_ (eqn (1)), and hence molecular alignment, the explanation for the appearance of TQF signals is more complicated as these signals can be detected in the absence of the molecular order, or when *ω*_eff_ = 0. The inspection of eqn (2) reveals that quadrupolar coupling, and therefore alignment, is indeed not the only source for TQF signals. An alternative cause for TQF signals can be a bi-exponential transverse relaxation that occurs when the relaxation rate constants *R*^2^_slow_ and *R*^2^_fast_ in eqn (2) are not equal. Generally, for bi-exponential relaxation in the nuclear spin 
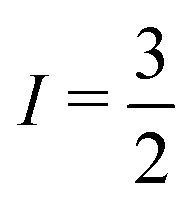
 system of ^23^Na to occur, the sodium ions need to be within a molecular environment that exhibits slow motion and that temporarily binds Na^+^ ions, thereby slowing also the motional dynamics of the sodium itself. Therefore, based on the absence of TQF signals when shear is not imposed, as shown in Fig. 5 (right panel) and Fig. 6(b), one can conclude that the macromolecules are not in an aligned phase, and furthermore, the molecules experience fast molecular tumbling that causes *R*^2^_fast_ = *R*^2^_slow_. The analysis of the TQF curves obtained under shear conditions in Fig. 6(b) indicates not only that quadrupolar coupling, and hence alignment is present, but also the bi-exponential relaxation occurs with significant differences between *R*^slow^_2_ and *R*^fast^_2_, suggesting a changed molecular dynamics of sodium ions under shear. The structure of this shear induced phase also changes with the increase of the applied shear rate as seen from the changed shape of both types of MQF signals displayed in Fig. 6(a and b).

**Fig. 7:**
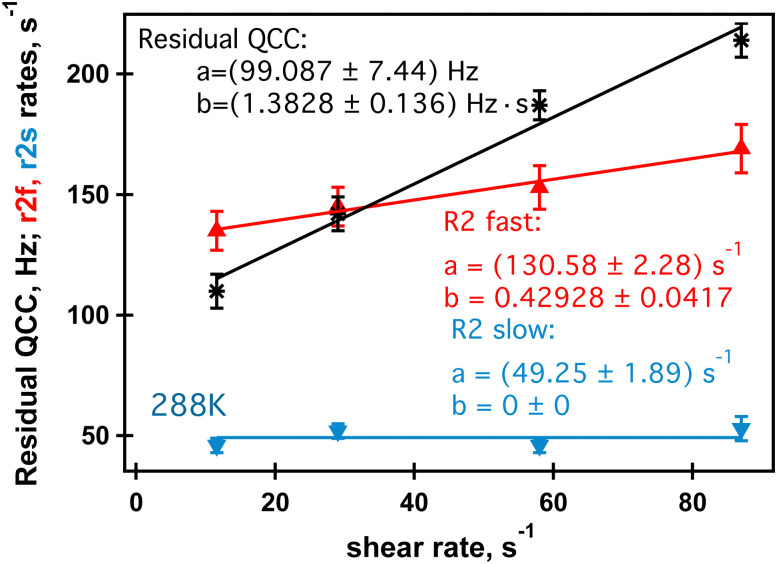
Sodium residual quadrupolar coupling constant (black stars), fast (red triangles) and slow relaxation (blue inverted triangles) in the shear induced phase formed in the 0.5% aqueous *κ*-carrageenan fluid at 288 K at different shear rates. Error bars represent errors reported in Table 2.

Further analysis of the shear dependence of sodium MQF signals indicates that the parameter *R*^slow^_2_ is independent of the applied shear rate while *ω*_eff_ increases linearly with the increase of the shear rate, Fig. 7. A slight shear rate dependence may also exist for *R*^fast^_2_, although this may require further study. However, a remarkable finding is that in the absence of shear, both, the coupling *ω*_eff_ and the bi-exponential nature of the relaxation disappear. While *ω*_eff_ = 0 indicates the absence of the alignment, an exponential transverse relaxation indicates the changed motional dynamics of the ions in the presence of the biomolecules. The changed dynamics of the sodium ions can be caused by a reduced overall tumbling of the molecules in the aligned phase. Alternatively, an increased density of macromolecules in the aligned phase may also alter the sodium dynamics because there are simply more interaction events for the ions in the vicinity of the molecules. This should also contribute to the bi-exponential relaxation observed in the TQF experiments, but the precise process will require further study. Note that sodium ions in other phases, if present, remain invisible for TQF spectroscopy.

**Fig. 8:**
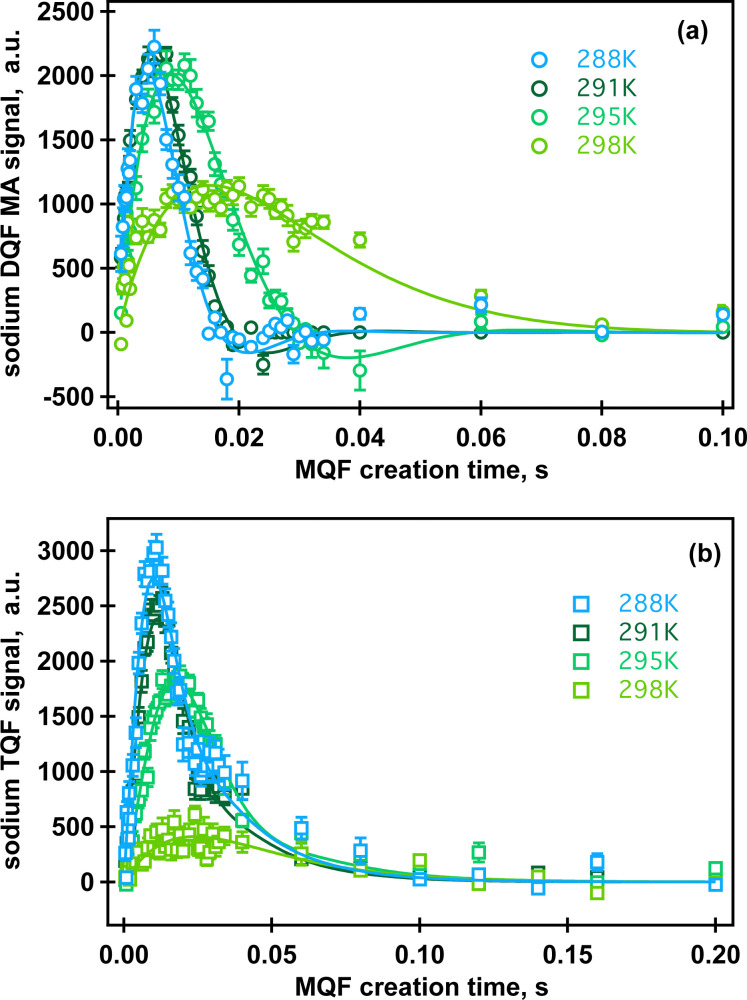
Multiple quantum response at *γ̇* = 58 s^−1^ and at 288 K–298 K: (a) ^23^Na DQFMA and (b) ^23^Na TQF time evolution. Solid lines represent data fitting to eqn (1) and (2) for the DQF MA and TQF data, respectively. Relevant fitting parameters are reported in Table 3; please refer to the ESI† for additional details.

The ^23^Na MQF rheo-NMR data of the 0.5% aqueous *κ*-carrageenan fluid collected in the extended temperature range and at *γ̇* = 58 s^−1^ are displayed in Fig. 8.

**Fig. 9:**
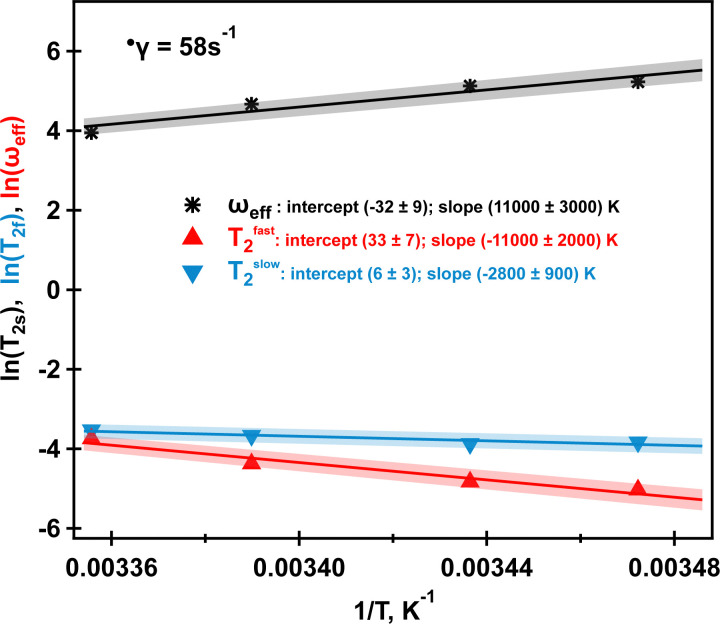
Arrhenius plot of *ω*_eff_ (black stars), *T*^fast^_2_ (red triangles) and *T*^slow^_2_ (blue inverted triangles) at *γ̇* = 58 s^−1^ for four studied temperatures. Solid lines represent linear fits with shading indicating a 5% range deviation from the predicted linear behaviour. Intercepts and slopes and their errors for each fit are shown in the legend. Further explanations are in the text. Data to produce the plot are taken from Table 3.

The results of the analysis of these data using both eqn (1) and (2) are reported in Table 3. The curve fit at 298 K can be found in Fig. SI6, ESI.† As at 288 K, similar MQF behaviour is observed in this fluid up to 298 K as shown in Fig. 8. Above 303 K no multiple quantum signals were observed neither in the presence nor in the absence of the applied shear field as shown in Fig. SI5, ESI.† We associate this behaviour with the onset of molecular phase transition from helices to random coils for the *κ*-carrageenan molecules in this temperature range. At 303 K, although *κ*-carrageenan molecules still may exist in the helix conformation, the helices most likely start to unfold as their elasticity probed by oscillatory rheology is rapidly decreasing. The application of shear at 303 K does not create a stable ordered phase that can be detected using sodium MQF methods, probably because of the increased molecular motion of sodium ions. At higher temperatures, it is unlikely that the macromolecules in the random coil conformation form a stable aligned phase; therefore, *ω*_eff_ = 0 and no DQF MA signals are detected. Furthermore, although the tumbling of the overall molecule may be reduced in the random coil conformation, the fast internal motion becomes possible that prevents bi-exponential transverse relaxation. Therefore, TQF signals are also no longer present, even under shear. Temperature dependent measurements provide further insights into sodium relaxation and the molecular alignment in the shear induced ordered phase. The Arrhenius plots of sodium 
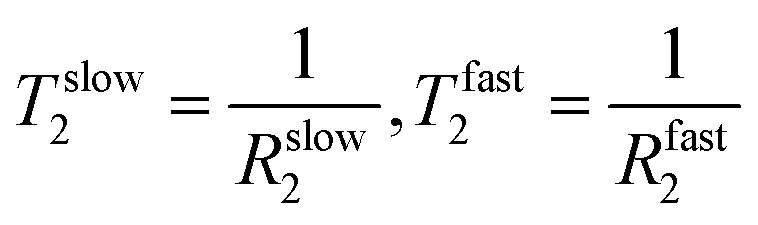
 and *ω*_eff_ at *γ̇* = 58 s^−1^ in the temperature range from 288 K to 298 K are displayed in Fig. 9. As one can see from Fig. 9, *T*^slow^_2_ appears to be largely independent on the inverse of temperature, while *T*^fast^_2_ demonstrates linear dependence on the inverse temperature with a negative slope. The observed behavior can be explained as follows. The molecular tumbling in the shear aligned phase is very much reduced by the intermolecular forces that keep the molecules aligned. The correlation times of these molecules are therefore very long. For ^23^Na^+^ relaxation, however, the relevant correlation times are dictated by adsorption and desorption events to and from the biomolecules in the aligned phase and are therefore best described by the Arrhenius relationship. As the temperature increases, the adhesion time of the sodium ions to the aligned phase is reduced, and therefore, the ^23^Na relaxation relevant correlation time is shortened. This leads to an increase in the observed relaxation time *T*^fast^_2_ as observed by the negative slope in the inverse temperature plot in Fig. 9. *T*^slow^_2_, unlike *T*^fast^_2_, does not depend on the spectral density at zero frequency, and hence deviates from this behavior, reminiscent to the well-known *T*_1_, *T*_2_ dipolar relaxation behaviour as a function of correlation time *τ*_c_. Furthermore, as the temperature increases, the *κ*-carrageenan helices start to gain additional degrees of freedom and do not align this readily in the shear field. Therefore, the residual quadrupolar coupling constant *ω*_eff_ also decreases during this process. It reaches a low value of *ω*_eff_ = 28 Hz at *T* = 298 K and this is probably also associated with the onset of the unfolding of *κ*-carrageenan helices and decreased the molecular alignment that can be probed by anisotropic sodium dynamics. Above this temperature, no alignment is detected upon imposition of the shear field and sodium dynamics becomes isotropic as only single quantum sodium signals are detected, Fig. 5. This correlates well with the bulk shear and oscillatory rheology data displayed in Fig. 2 that also captures changes in the fluid associated with fluid's molecules transitioning from helices to random coils upon the temperature ramp.

**Table 3:** Relevant parameters extracted after fitting the data displayed in Fig. 8 using eqn (1) and (2); please refer to Table SI4, ESI, for additional fitting details

Temperature, K	*ω*_eff_, Hz	*R* ^fast^_2_, s^−1^	*R* ^slow^_2_, s^−1^
288	188 ± 6	153 ± 9	46 ± 3
291	163 ± 4	136 ± 5	48 ± 3
295	107 ± 2	79 ± 4	39 ± 3
298	52 ± 1	42 ± 1	34 ± 2
303	—	—	—
313	—	—	—

## 4 Conclusions

A molecular–mechanical correlation was found for the 0.5% *κ*-carrageenan fluid with evidence of the shear-induced ordered self-assembly of *κ*-carrageenan helices in the fluid. Any evidence of ordered self-assembly was absent in the absence of shear and when *κ*-carrageenan molecules were in the random coil conformation at high temperatures. The mechanical properties of the fluid confirmed that the viscoelasticity of the fluid was lost upon temperature ramp and the fluid became Newtonian upon complete transition from helices to random coils. The loss of shear-induced self-assembly in this case was clearly captured using ^23^Na MQF rheo-NMR methods. This particular model system, which displays these mechanics without having an explicit liquid crystalline phase, underlines the potentially higher impact of ^23^Na rheo-NMR for many biologically relevant fluids. For example, synovial fluid, blood and various polysaccharide solutions used in drug delivery contain naturally present Na^+^ cations that may potentially manifest the shear-induced structure without having an explicit liquid crystalline phase. The present work considered endogenous sodium levels (0.02%) that are almost 45 times lower than the physiological sodium concentration in the body (0.9%). Hence, *in vivo* monitoring of the underlying shear-induced structure of body fluids might be accomplished *in vivo* and may perhaps be correlated with clinical outcomes. Moreover, most human scanners are capable of operating at the sodium frequency; therefore, natural sodium could be used as a tracker to non-invasively capture changes in the microstructure of body fluids and tissues associated with clinical conditions.

## Author contributions

GEP designed experiments and performed the data collection and analysis. GEP and TM co-wrote the paper.

## Conflicts of interest

There are no conflicts to declare.

## Supplementary Material

SM-019-D2SM01381A-s001
