# Supplementary material for: A molecular–mechanical link in shear-induced self-assembly of a functionalized biopolymeric fluid
Source: Soft Matter. 2023 Apr 3;19(17):3228–37. doi: 10.1039/d2sm01381a (PMC10155600; doi:10.1039/d2sm01381a)
Supplement: SM-019-D2SM01381A-s001 [file SM-019-D2SM01381A-s001.pdf]

Electronic Supplementary Material (ESI) for Soft Matter.
This journal is © The Royal Society of Chemistry 2023

Electronic Supplementary Information.

Molecular-mechanical link in shear-induced self-assembly of a functionalized biopolymeric fluid.

Galina E Pavlovskaya[a,b],* *and Thomas Meersmann* [a,b]
[a]Sir Peter Mansfield Imaging Resonance Centre, School of Medicine, University of Nottingham, Nottingham, NG2 7RD, United Kingdom. Tel: +44115 84 68131;
[b]NIHR Nottingham Biomedical Research Centre, Nottingham, NG2 7RD, United Kingdom.
E-mail: galina.pavlovskaya@nottingham.ac.uk

Flow rheology.

Table SI1. Additional experimental details of multiple flow experiments.

Temperature, K	Lower shear rate limit, s^{-1}	Upper shear rate limit, s^{-1}	Equilibration time, s	Single point measurement delay, s	Single point measurement sampling time, s	Repeats
283	1e-04<br>1e-03	200<br>200	600<br>120	5<br>5	30<br>30	2
288	1e-04<br>1e-04<br>1e-04<br>1e-03	200<br>200<br>200<br>200	120<br>120<br>120<br>600	5<br>5<br>5<br>5	30<br>30<br>30<br>30	4
295	0.1<br>1e-3<br>0.01<br>0.01	200<br>200<br>200<br>200	600<br>120<br>120<br>120	5<br>5<br>5<br>5	30<br>30<br>30<br>30	4
303	1e-4<br>1e-3<br>1e-4	200<br>200<br>200	120<br>120<br>120	5<br>5<br>5	30<br>30<br>30	3
313	0.1<br>1e-4<br>1e-4	200<br>200<br>200	120<br>120<br>120	5<br>5<br>5	30<br>30<br>30	3

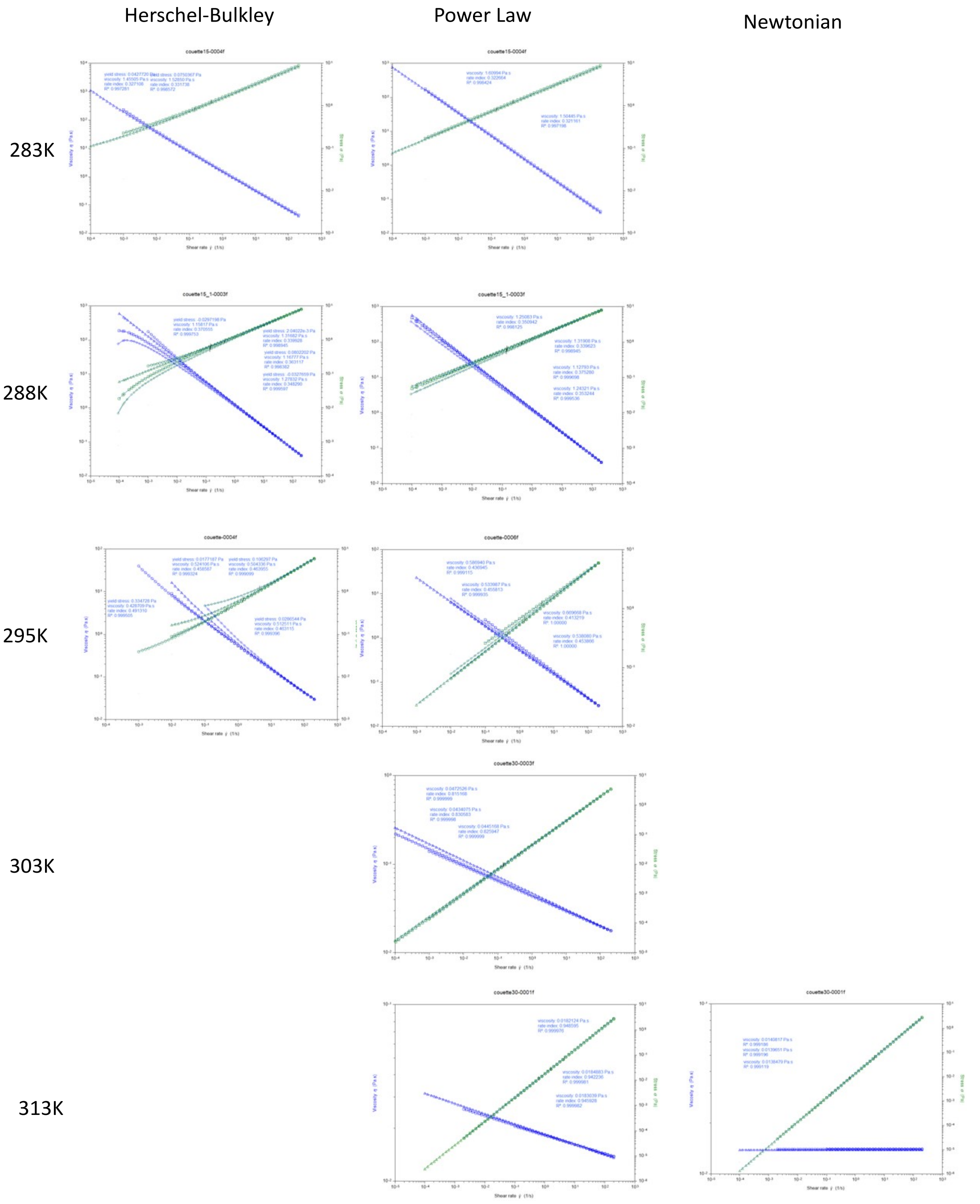

Figure SI1 Workflow of fluid model assignment based upon TRIOS analysis.

Table SI2. Parameters with SDs for all models tried determined from the analysis of the replicated flow data sets.

Temperature, K	Yield stress, Pa	Viscosity, Pa.s, HB	Viscosity, Pa.s, Power law	Viscosity, Pa.s, Newtonian	Rate index, n HB	Rate index n, Power law
283	0.06 ± 0.02	1.49 ± 0.05	1.56± 0.07	-	0.35 ± 0.03	0.322 ± 0.001
288	0.02 ± 0.07	1.23 ± 0.08	1.24 ± 0.08	-	0.36 ± 0.01	0.35 ± 0.01

295	0.1 ± 0.2	0.49 ± 0.04	0.58 ± 0.06	-	0.47 ± 0.01	0.44 ± 0.02
303	-	-	0.0451 ± 0.002	-	-	0.824 ± 0.006
313	-	-	0.018 ± 0.001	0.0140 ± 0.0001	-	0.946 ± 0.002

Oscillatory rheology.

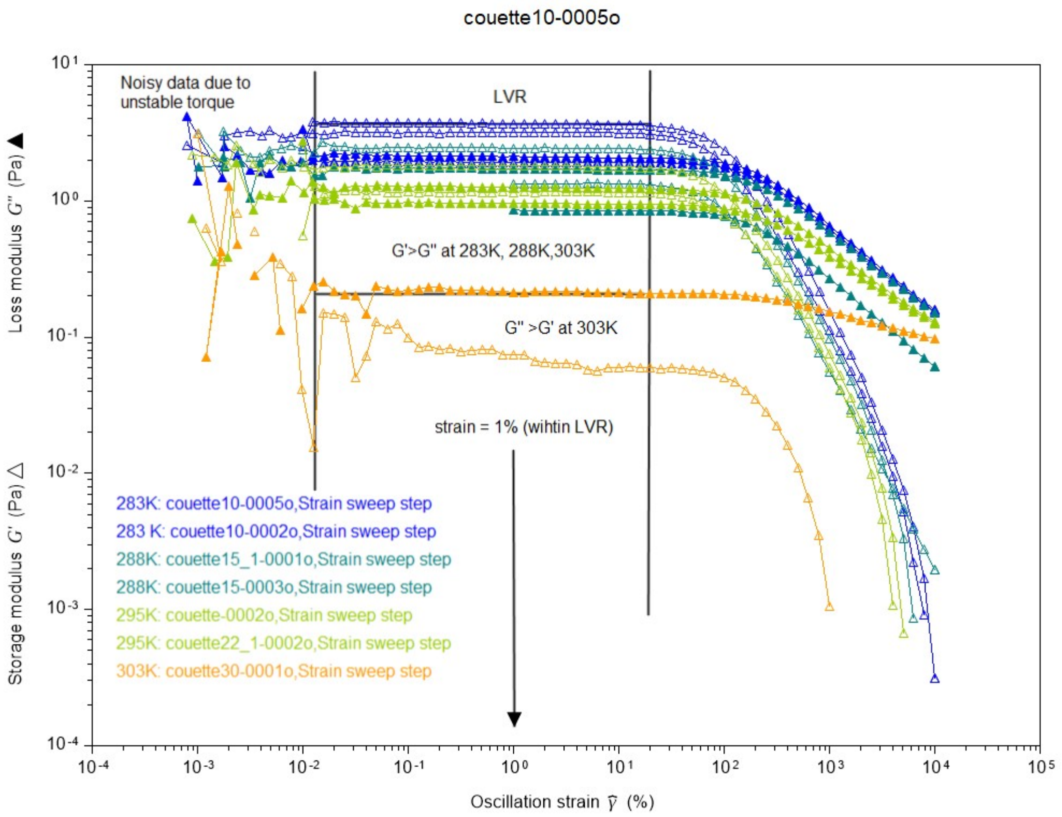

Figure SI2. Replicated amplitude sweeps at ω=6.28 rad/s at 283K, 288K, 295K and 303K. Only one amplitude sweep was collected at 303K because G" became large than G'. Strain sweeps at 313K were not performed as the fluid became Newtonian. LVR region was determined as shown in the plot.

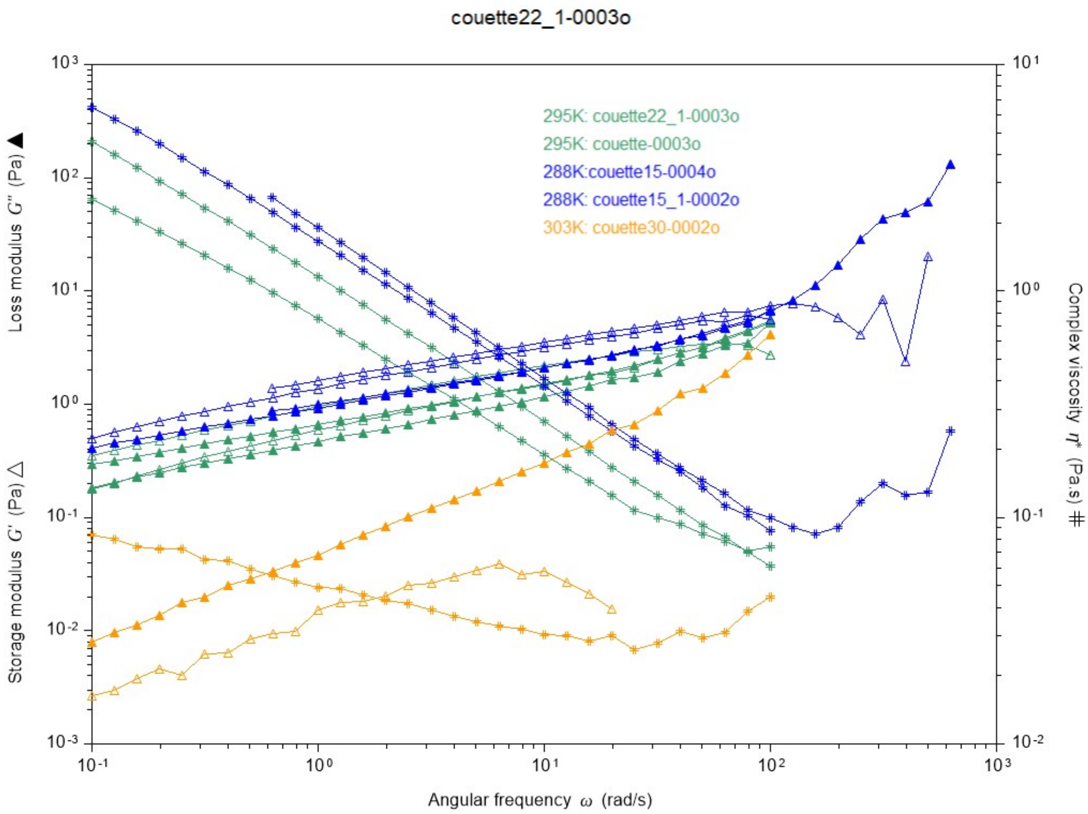

Figure SI3. Duplicates of frequency sweeps at 1% strain. Complex viscosity was evaluated using built-in Cox-Merz transformation. The quality could be improved if a higher strain within the LVR was used. These data were used in the main text.

Rheo-NMR.

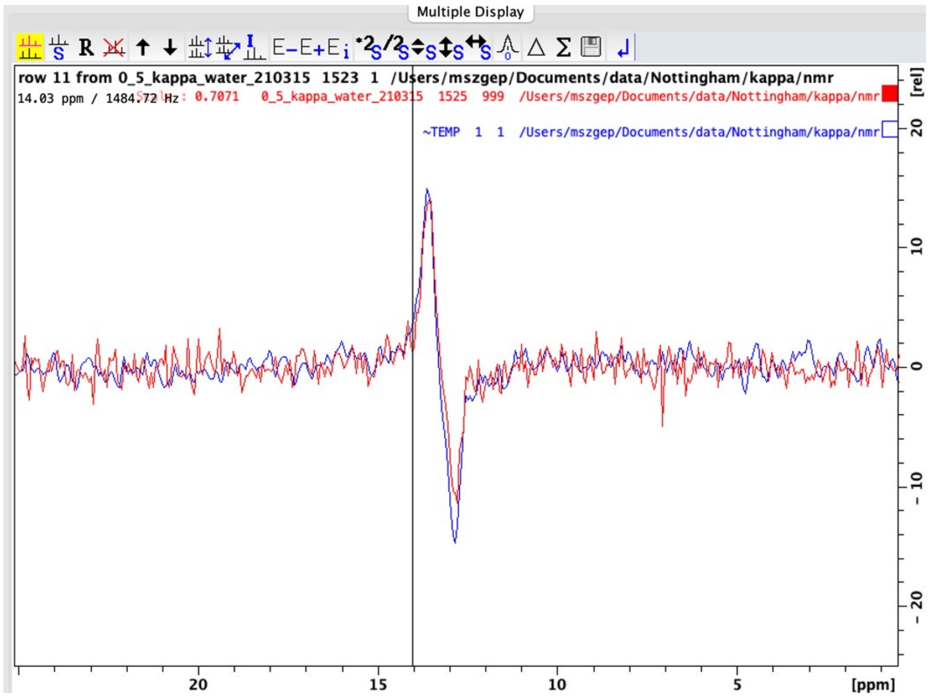

Figure SI4. Replicated spectra used to produce replicated data points shown In Fig. 6(a) main text.

Table SI3. C0 and C1 amplitudes at 288K as a function of shear rate.

Shear rate at 288K, s^{-1}	*C0*	*C1*
11.6	6512.4 ± 620	4075.4 ± 216
29	5977.3 ± 446	4363.4 ± 207
58	5467.7 ± 338	3963.3 ± 168
87	6149.6 ± 382	4972.4 ± 342

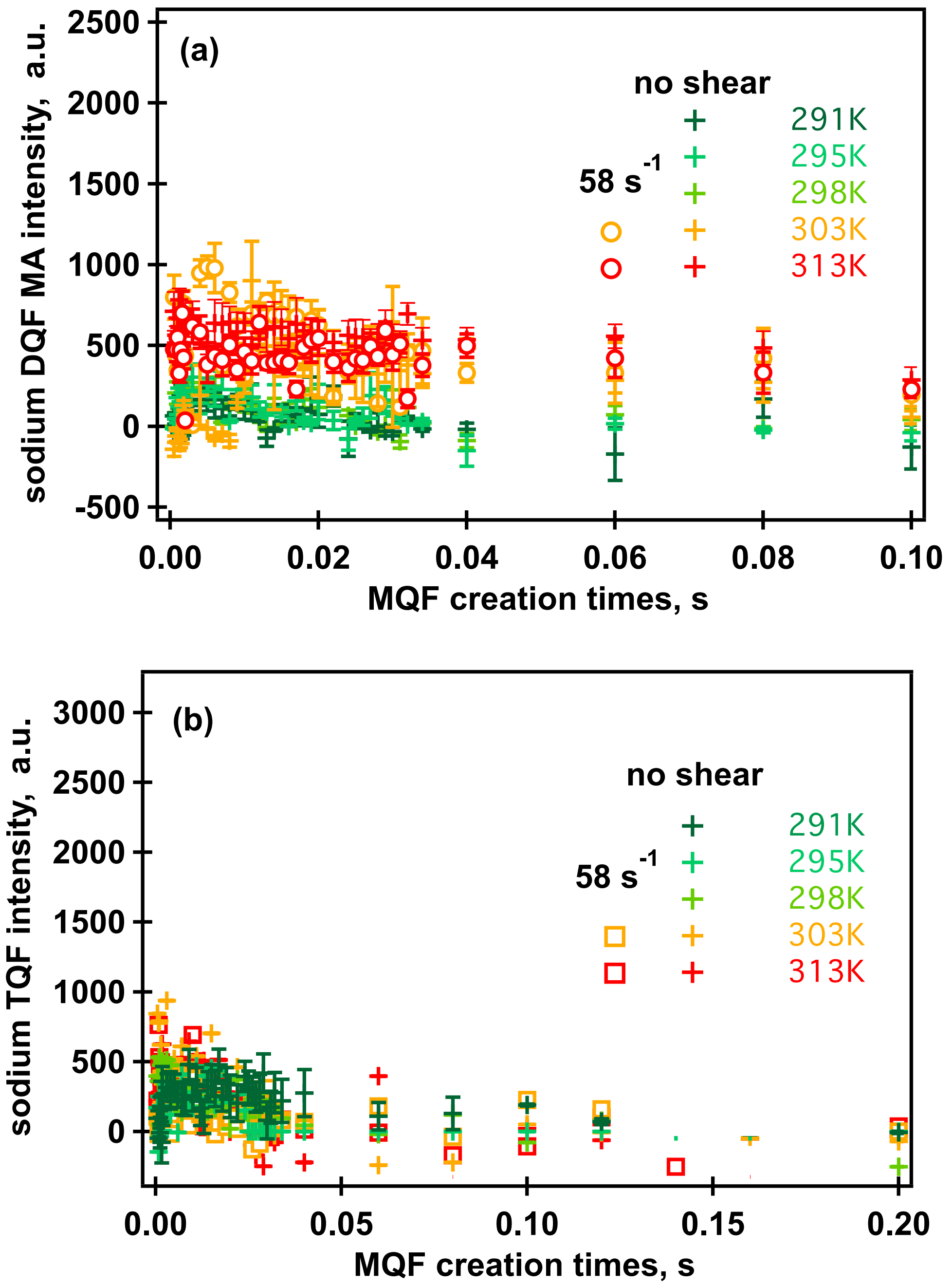

Figure SI5. Multiple Quantum Response at the shear rate = 58s $^{-1}$ at 303K, 313K and in the absence of shear in the extended temperature range: (a) - ^{23}Na DQFMA and ^{23}Na TQF time evolution. No fitting was performed for data collected at 303K and

313K due to the scatter in τ dependencies of both types of MQF signals. Data collected in the absence of shear are shown in crosses. Note that where fitting could not be performed with the satisfactory errors, the data under shear followed similar trend as the data collected in its absence.

Table SI4. C0 and C1 amplitudes at 58s^{-1} as a function of temperature.

Temperature, K	*C0*	*C1*
288	5468 ± 338	3963 ± 168
291	4507 ± 389	3621.7 ± 152
295	4983 ± 230	3120.2 ± 172
298	2972 ± 95	1202 ± 102

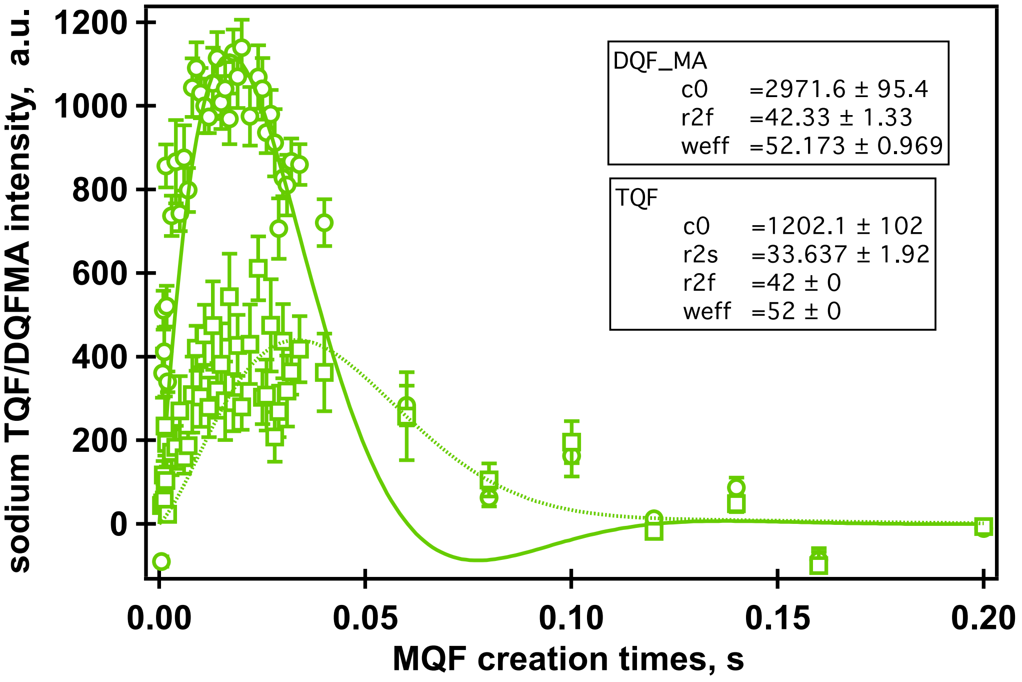

Figure SI6. A graph returned after the data analysis at 298K by IGORPro 8.0 (Wavemetrics, USA.) software. DQF MA and TQF data are shown in circles and squares, respectively. Predicted DQF MA and TQF dependencies are shown in solid and dashed lines, respectively.
